# Novel computational analysis of large transcriptome datasets identifies sets of genes distinguishing chronic obstructive pulmonary disease from healthy lung samples

**DOI:** 10.1038/s41598-021-89762-8

**Published:** 2021-05-13

**Authors:** Fabienne K. Roessler, Birke J. Benedikter, Bernd Schmeck, Nadav Bar

**Affiliations:** 1 Department of Chemical Engineering, Norwegian University of Science and Technology (NTNU), Trondheim, Norway; 2 Institute for Lung Research, Universities of Giessen and Marburg Lung Centre, Philipps University Marburg, Marburg, Germany; 3 Department of Medical Microbiology, Maastricht University Medical Center (MUMC+), Maastricht, The Netherlands; 4 Department of Pulmonary and Critical Care Medicine, University Medical Center Marburg, Universities of Giessen and Marburg Lung Center, Philipps University Marburg, Hesse, Germany; 5 Institute for Lung Health (ILH), Giessen, Germany; 6 Member of the German Center for Lung Research (DZL), the German Center for Infection Research (DZIF), and the Center for Synthetic Microbiology (SYNMIKRO) Marburg, Hesse, Germany

**Keywords:** Chronic obstructive pulmonary disease, Bioinformatics, Microarray analysis, Computational biology and bioinformatics

## Abstract

Chronic obstructive pulmonary disease (COPD) kills over three million people worldwide every year. Despite its high global impact, the knowledge about the underlying molecular mechanisms is still limited. In this study, we aimed to extend the available knowledge by identifying a small set of COPD-associated genes. We analysed different publicly available gene expression datasets containing whole lung tissue (WLT) and airway epithelium (AE) samples from over 400 human subjects for differentially expressed genes (DEGs). We reduced the resulting sets of 436 and 663 DEGs using a novel computational approach that utilises a random depth-first search to identify genes which improve the distinction between COPD patients and controls along the first principle component of the data. Our method identified small sets of 10 and 15 genes in the WLT and AE, respectively. These sets of genes significantly (*p* < 10^–20^) distinguish COPD patients from controls with high fidelity. The final sets revealed novel genes like cysteine rich protein 1 (CRIP1) or secretoglobin family 3A member 2 (SCGB3A2) that may underlie fundamental molecular mechanisms of COPD in these tissues.

## Introduction

Chronic obstructive pulmonary disease (COPD) is characterised by persistent respiratory symptoms (shortness of breath, cough, sputum production) and airflow limitation originating from a mixture of small airways disease and parenchymal destruction (emphysema)^1^. The disease is caused by the exposure to noxious particles and gases with cigarette smoking as the main risk factor, especially in high-income countries^1,2^. In 2015, 3.2 million people died of COPD, making it the 3rd leading cause of death worldwide after ischaemic heart disease and stroke^3,4^. Despite the global burden of COPD, which is predicted to increase further in the following years^5,6^, knowledge regarding its pathogenesis and underlying molecular mechanisms remains limited. A better understanding of these mechanisms could potentially lead to new targets for prevention, treatment and prognosis of COPD.

One possibility to investigate differences in molecular mechanisms between COPD patients and a control population without COPD is to perform gene expression analyses of clinically collected lung samples. Such studies have previously been performed both on samples from the whole lung tissue (WLT)^7–12^ and the airway epithelium (AE)^13–15^ of COPD and control subjects, revealing different sets of differentially expressed genes (DEGs) associated with the disease. One difficulty of expression analysis is the large resulting number of DEGs, which normally ranges between 100 and 300, making an in-depth analysis of every single gene too challenging. Common ways to select key important COPD-associated DEGs are to focus on the strongest regulated genes or to select them based on an association with biological functions which potentially are disease-relevant. A recent study by Mostafaei et al. pre-selected 90 genes associated with the progression of COPD. To further reduce the set of genes, different machine learning-based and statistical methods were tested and reduced the set to a manageable 44 genes associated with COPD or lung function^14^.

Another common drawback of gene expression studies in COPD is a limited access to a large number of lung samples. Some of the reported COPD studies had access to only a low number of patients (i.e. lung samples)^7–9^, originating from the highly invasive nature of collecting such samples. Gene expression analyses based on low numbers of patient samples are at risk in underestimating the heterogeneity of such samples, partially leading to the poor replicability of the resulting sets of genes between studies^16^. One manner to increase the sample size is by combining several independent gene expression datasets as has been done by previous studies^12,15^. Careful scrutiny of the different datasets should then be taken to account for variations in age, health status, smoking status, the presence of comorbidities, etc. Nevertheless, this approach can remove variability which stems from different sampling methods, geographical and ethnical differences, among others.

Here, we combined several public gene expression datasets to create a large transcriptome dataset containing WLT and AE samples from 405 and 411 subjects, respectively. Each dataset was tested for DEGs, which were only included for further analysis if they showed a consistent occurrence and the same trend of regulation in several datasets of the same lung sample type. These two criteria increase the likelihood that the remaining genes are associated with COPD in all the tested populations. We further showed that the resulting sets of 436 and 663 DEGs can be used to distinguish COPD from control subjects using principal component analysis (PCA). To decrease the number of COPD-associated DEGs, we developed a novel approach combining PCA with a random depth-first search (RDFS). This approach randomly removes single genes while testing, using the first principle component, for an improvement in distinction between COPD and control subjects. In contrast to other black-box, machine-learning based methods, our gene set reduction method is based on a computational search-tree approach that locally maximises the distinction between COPD and control subjects using the first principal component, making the results easily interpretable. The method identified two sets of 10 and 15 genes (for the WLT and AE, respectively), which persistently appeared in all the resulting sets of discriminatory DEGs. Most of these 25 genes have not been previously associated with COPD and therefore reveal new potential players in the underlying molecular mechanisms of the disease.

## Results

### Included datasets and subject characteristics

We included five different GEO gene expression datasets in our analysis. These microarray datasets contained in total 405 and 411 subjects sampled from the WLT and the AE (bronchial and small AE (SAE)), respectively. We matched all COPD and control subjects of a dataset for the type of microarray used and their current smoking status (former smoker (FS) or current smoker (CS)). The latter means that statistical comparison was only conducted between COPD and control subjects with the same smoking status as this should ensure that DEGs emerge based on the disease status and not the differences in smoking status. This matching resulted in a list of seven comparison groups (WLT1-3 and AE1-4) (Table 1). Statistical testing showed that all comparison groups were matched for age and gender (*p* > 0.05) except for AE3 (age: *p* < 10^–4^) and AE4 (age: *p* < 10^–5^). The comparison groups AE1-3 were also matched for pack-years (*p* > 0.05), while WLT1 and AE4 showed a significant difference (*p* < 0.01) in smoking history between COPD subjects and controls. For WLT2 and WLT3, no information regarding smoking history was available. The forced expiratory volume in 1 s (FEV1) % predicted was for all comparison groups lower in COPD subjects compared to controls.

**Table 1:** Overview of the included GEO gene expression data sets and their subject characteristics.

	WLT						AE							
							Bronchial AE				SAE			
GEO Accession (country of sampling)	GSE76925^11^ (USA)		GSE47460 (USA)				GSE37147^13^ (Canada)				GSE11906^17^ (USA)		GSE64614^18^ (USA)	
Microarray platform	GPL10558		GPL6480		GPL14550		GPL6244		GPL6244		GPL570		GPL570	
Smoking status	FS		FS		FS		FS		CS		CS		CS	
Subject group	COPD	Control	COPD	Control	COPD	Control	COPD	Control	COPD	Control	COPD	Control	COPD	Control
Number of subjects	111	40	66	9	125	54	57	82	30	69	20	44	36	73
Mean age (± SD)	63.6 (± 6.6)	65.7 (± 9.0)	62.7 (± 10.8)	66.1 (± 10.2)	66.1 (± 9.1)	65.9 (± 10.4)	66.1 (± 5.6)	65.8 (± 5.0)	63.2 (± 6.7)	62.2 (± 6.0)	52.1 (± 8.1)	43.5 (± 5.9)	*52*^*a*^ *(*± *7)*	*43* *(*± *8)*
Male/female	52/59	15/25	41/25	6/3	69/56	30/24	36/21	49/33	16/14	34/35	16/4	31/13	*28/9*^*a*^	*53/20*
Pack-years (± SD)	*61.3* *(*± *26.3)*	*33.6* *(*± *21.0)*	–	–	–	–	52.9 (± 28.1)	48.3 (± 22.9)	47.5 (± 13.9)	44.3 (± 11.2)	37.6 (± 23.4)	28.6 (± 16.4)	*38*^*a*^ *(*± *24)*	*27 (*± *16)*
FEV1% predicted (± SD)	26.5 (± 9.4)	98.7 (± 12.5)	55.5 (± 26.8)	107.7 (± 11.2)	53.7 (± 21.9)	96.6 (± 8.7)	61.6 (± 12.1)	93.1 (± 13.1)	57.6 (± 16.4)	92.2 (± 13.7)	*72* *(*± *22)*	*109* *(*± *14)*	*82*^*a*^ *(*± *21)*	*107 (*± *14)*
Name of comparison group	WLT 1^c^		WLT 2		WLT 3		AE 1		AE 2		AE 3^b^		AE 4^b,c^	

*Cursive values* were taken from the original publications and not calculated by us.

^a^The values displayed were taken from the original publication and include an additional COPD subject which was not included in our study.

^b^The comparison group is not age matched (*p* < 10^–4^).

^c^The comparison group is not matched for pack-years (*p* < 0.01).

### Selecting COPD-associated DEGs based on consistent occurrence and same sign of log2 fold change

**Figure 1:**
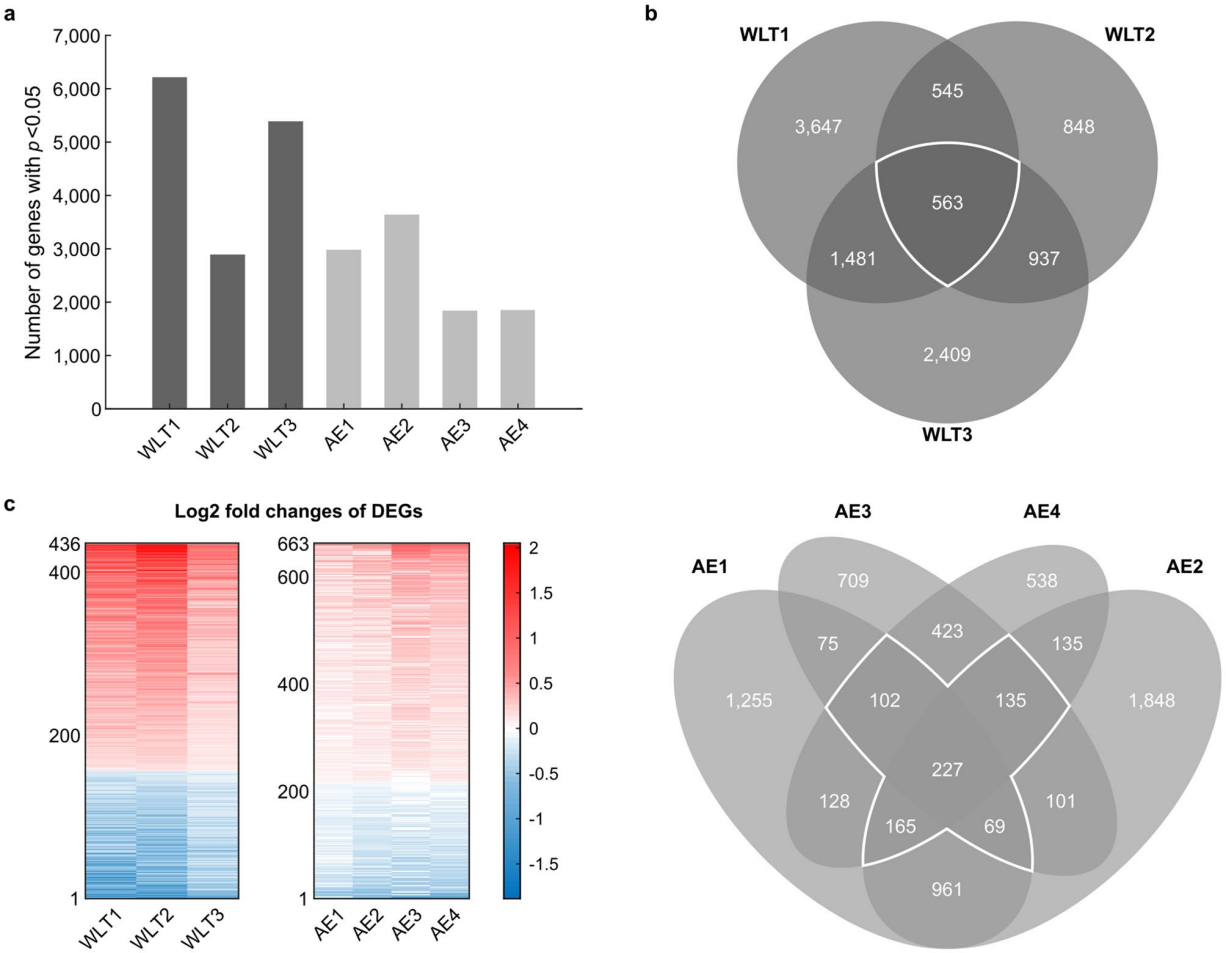
Selection of COPD-associated DEGs for the WLT and the AE. (**a**) Bar diagram showing the number of significant (*p* < 0.05) DEGs found in each comparison group. (**b**) Venn diagrams showing the overlap of DEGs between different comparison groups. We only compared groups of the same lung sample type. White-outlined sections mark DEGs that fulfil the first selection criteria (see Results) for COPD-associated DEGs. (**c**) Heatmap showing fold changes of the 436 and 663 COPD-associated DEGs from the WLT and the AE, respectively. The DEGs were sorted by the mean fold change over all comparison groups.

Each comparison group was processed and tested for significant DEGs separately. In total, 17,249 genes were measured and tested in each group. The statistical testing revealed different numbers of significant (*p* < 0.05) DEGs for each comparison group varying between 1841 (AE3) and 6236 (WLT1) genes (Fig. 1a). To increase our confidence of a DEG being associated with COPD in all the populations we tested, we selected DEGs based on the combination of the following two criteria:

1. Significant (*p* < 0.05) DEG in three or more comparison groups of the same lung sample type (white outlined in Fig. 1b).
2. Same log2 fold change sign over three or more comparison groups of the same lung sample type.

Selection criterion one assures that the selected DEGs reproducibly emerge in several independently tested populations. Criterion two assumes that DEGs are affected by COPD in a similar manner across all the populations. This excludes genes that are up-regulated in one comparison group, but down-regulated in another and vice versa. 436 DEGs and 663 DEGs satisfied these two criteria in the WLT and the AE, respectively. In both sample types, about two third of the DEGs were upregulated (WLT: 279/436 DEGs, AE: 446/663 DEGs) in COPD subjects compared to controls, while one third was downregulated (WLT: 157/436 DEGs, AE: 217/663 DEGs) (Fig. 1c). 34 of these DEGs satisfied the two criteria in both lung sample types.

### Distinguishing COPD from control subjects by applying PCA to expression values of COPD-associated DEGs

**Figure 2:**
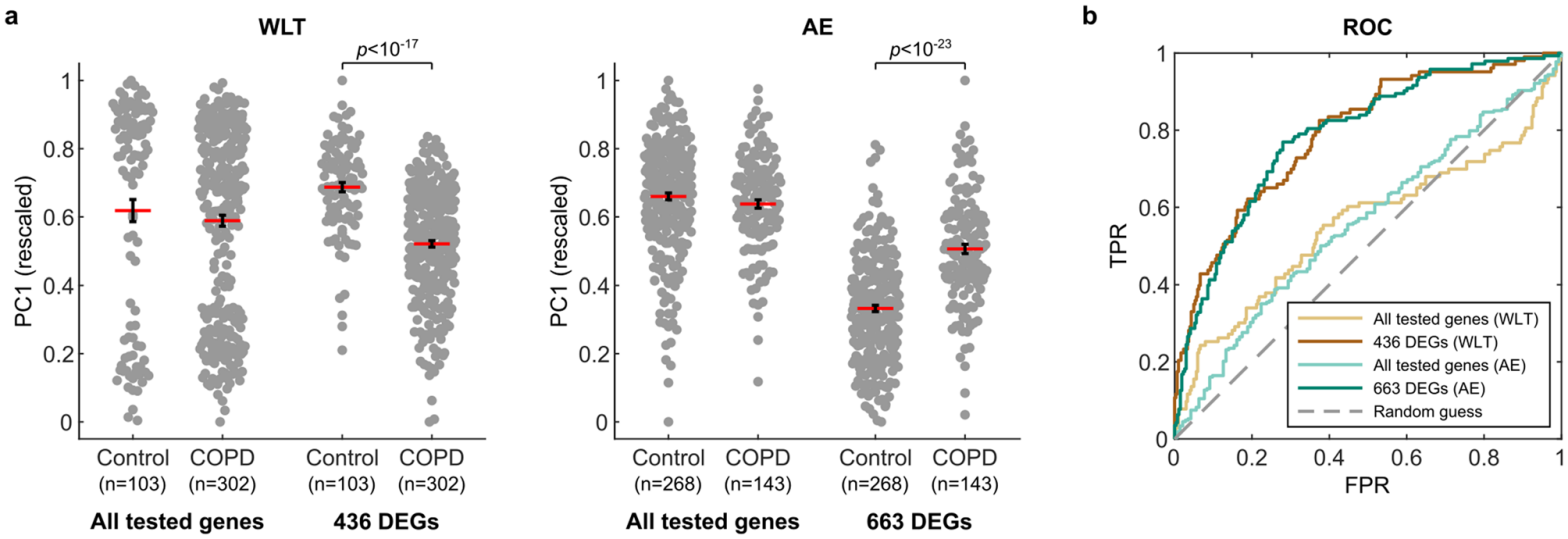
Distinction between COPD and control subjects using PC1. (**a**) Beeswarm plots comparing rescaled PC1 scores of control and COPD subjects for both lung sample types (WLT and AE). The PC1 scores are computed from either the gene expression values of all tested genes (17,249) or the corresponding COPD-associated DEGs. Only the COPD-associated DEGs lead to a significant difference (*p* < 0.05) between COPD and control subjects. Red lines with black error bars show the mean ± SEM. (**b**) ROC curves comparing the performance of the computed PC1 scores (see (**a**)) in distinguishing COPD from control subjects.

Based on our two selection criteria, we assumed that the two sets of DEGs are strongly associated with COPD and should distinguish COPD subjects from controls. To test this assumption for each lung sample type, we first combined the COPD and control subjects of the respective comparison groups into a single dataset (see Methods). When running a PCA on the rescaled expression values of the 436 DEGs from the WLT, we found that principal component 1 (PC1) described 29% of the variance in the data (Supplementary Figure S1). Importantly, there was a significant (*p* < 10^–17^) difference between the rescaled PC1 scores of COPD (mean: 0.52) and control subjects (mean: 0.69) (Fig. 2a, left). This distinction does not appear in the results of the PCA applied to all the 17,249 tested genes. Similarly, running the PCA on the 663 selected DEGs of the AE, PC1 described 25% of the variance in the data (Supplementary Figure S1) with a significant (*p* < 10^–23^) difference between the PC1 scores of the COPD subjects (mean: 0.51) and the controls (mean: 0.33) (Fig. 2a, right). We did not observe that the distinction between COPD and control subjects in PC1 emerged from the different comparison groups (e.g. WLT1 vs. WLT2 or AE2 vs. AE3), indicating that the rescaling of the data and the two selection criteria were successful in reducing larger batch effects in PC1 (Supplementary Figure S2). Nonetheless, some variance in gene expression remained from the differences in comparison groups (see PC2 in Supplementary Figure S2, middle column). The differences in PC1 scores led to a good performance in distinguishing COPD from control subjects for both sets of COPD-associated DEGs compared to all tested genes (Fig. 2b). The area under the ROC curve (AUC) for the two sets of 436 and 664 DEGs was 0.79, while it remained close to a random guess for all tested genes (WLT: AUC = 0.55, AE: AUC = 0.56). Thus for the COPD-associated DEGs, PC1 is an indication for the distinction between COPD and control subjects.

### Reducing the number of COPD-associated DEGs while improving distinction between COPD and control subjects using a RDFS

**Figure 3:**
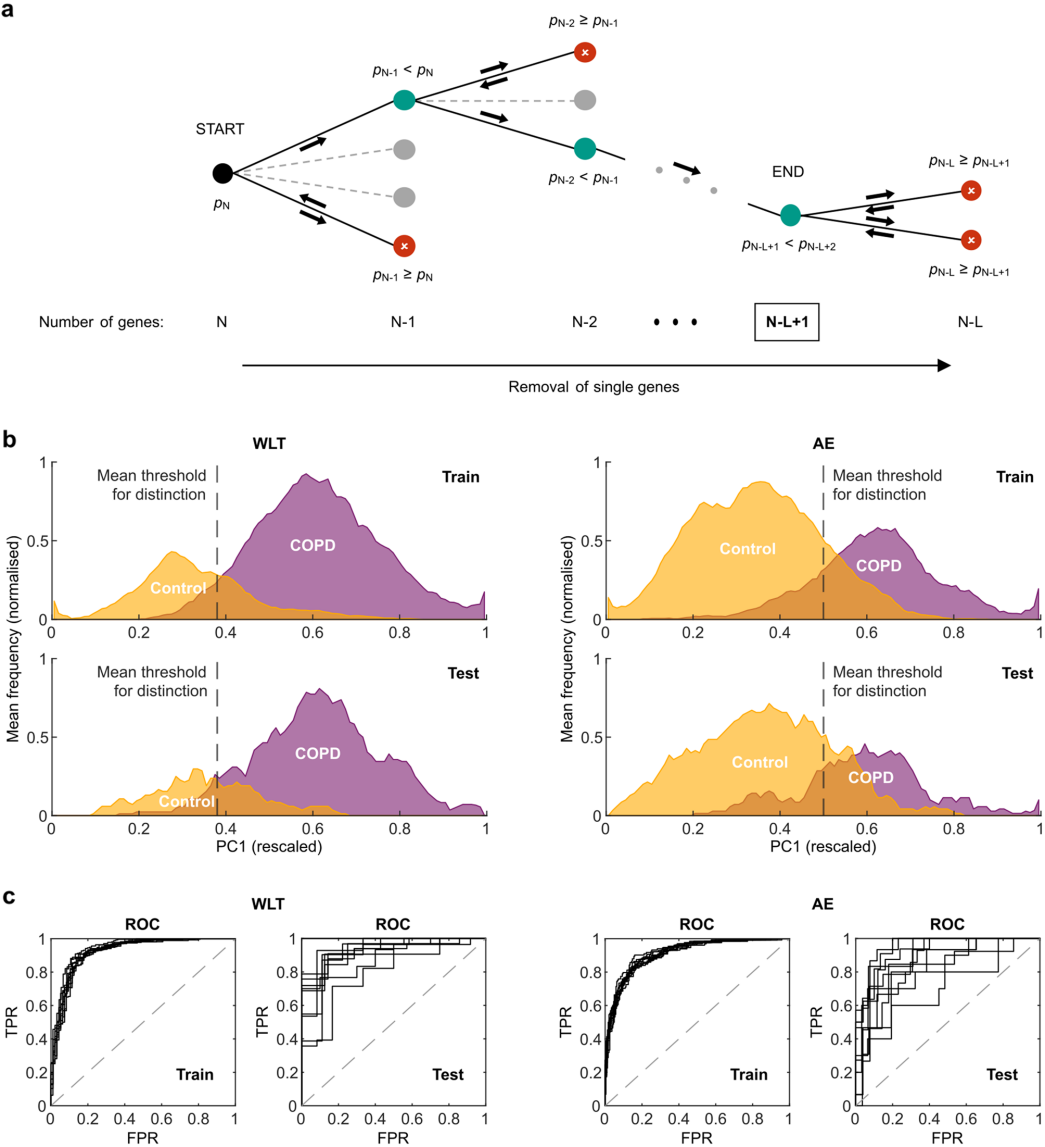
Search for small sets of discriminatory DEGs. (**a**) Visualisation of one iteration of our RDFS approach. The search starts with the full number of COPD-associated DEGs (= N) on the left and continues to the right (black arrows) by randomly removing single genes. After a removal, the PC1 scores for the two subject groups are computed from the expression values of the remaining subset of DEGs (e.g. N − 1) and the *p* value using a *t*-test is calculated. If the newly calculated *p* value is smaller than the previous one (e.g. *p*_N−1_ < *p*_N_), the gene is removed entirely, and the search continues on that branch of the search tree by randomly removing another gene. If the *p* value is equal to or bigger than the previous one (e.g. *p*_N−1_ ≥ *p*_N_), the gene is returned to the set of DEGs and another random gene is removed and tested. The search ends if no removal of a gene leads to a decrease in *p* value and the remaining subset of DEGs (N − L + 1, with L = depth of search tree) is the smallest set of discriminatory DEGs for this iteration. (**b**) Smoothed histograms showing the mean frequency of rescaled PC1 scores for COPD and control subjects over all ten search runs. The top ones consider only training subjects, while the lower ones consider only test subjects. The dashed line represents the mean threshold with the highest F-scores in distinguishing COPD from control subjects. (**c**) ROC curves showing the performance of the different sets of discriminatory DEGs in distinguishing COPD from control subjects. The dashed line represents the performance of a random guess.

To test if a reduced number of COPD-associated DEGs can improve the distinction between control and COPD subjects, we used a combination of PCA and a RDFS approach. By randomly removing single genes, the RDFS searches for subsets of DEGs that improve the distinction between COPD and control subjects using PCA (Fig. 3a). The searches were performed on 90% of the total number of subjects (training), while the validation of the performance was conducted on the remaining 10% of subjects (test).

Our RDFS approach resulted for both lung sample types in a strong reduction of the number of COPD-associated DEGs. For the WLT, the number over all ten search runs decreased from 436 to 35 (± 5.62) DEGs on average (Supplementary Table S1). For the AE, the number of DEGs was reduced from 663 to 66 (± 4.20) (Supplementary Table S2). These sets of discriminatory DEGs all resulted in a clear distinction between COPD and control subjects using PC1 for both the training and test subjects (Fig. 3b and Supplementary Figures S3, S4, S5). Using again ROC curves to assess the performance, the sets of discriminatory DEGs for both lung sample types achieved a mean AUC above 0.9 for the training subjects (Fig. 3c and Supplementary Tables S1, S2). In the case of the WLT, a similar performance was achieved on the test subjects, while for the AE, the AUC were on average slightly lower (0.87 ± 0.07) (Fig. 3c and Supplementary Table S2). Comparing these performance measures to the initial sets of COPD-associated DEGs, the small sets of discriminatory DEGs for both lung sample types indeed improved the distinction between COPD and control subjects.

### Persistent set of 10 and 15 DEGs remains during reduction process

**Figure 4:**
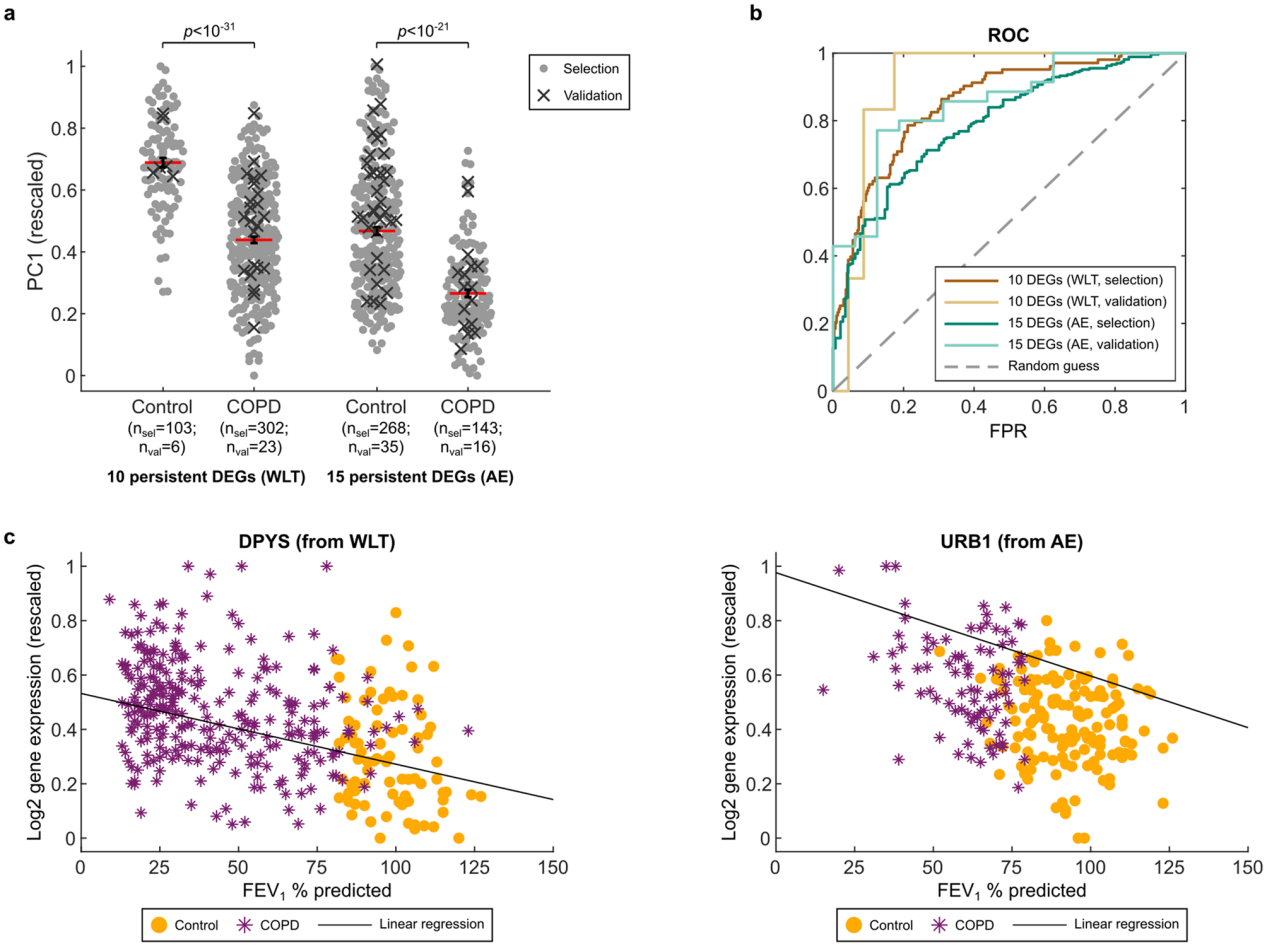
Study of 10 and 15 persistent DEGs. (**a**) Beeswarm plots comparing rescaled PC1 scores computed for COPD and control subjects using only the expression values of the two persistent sets of COPD-associated DEGs. For subjects used to select the persistent DEGs, the scores of the COPD subjects are significantly (*p* < 10^–20^) different compared to the control subjects. The PC1 scores computed for the validation subjects show their distribution in comparison to the subjects used for selection. Red lines with black error bars show the mean ± SEM (computed only for selection subjects). (**b**) ROC curves comparing the performance of the persistent DEGs in distinguishing COPD from control subjects using PC1 scores (see (**a**)). (**c**) Scatter plots showing the relation between the FEV_1_% predicted and the gene expression of DPYS in the WLT and URB1 in the AE of COPD and control subjects. The linear regression models the decrease in expression of DPYS and URB1 based on the increase in lung function, adjusted for comparison group, age and gender for DPYS or adjusted for smoking status (FS or CS), age, gender and pack-years for URB1.

During our search for smaller sets of COPD-associated DEGs, 10 and 15 DEGs persistently remained in the sets of discriminatory DEGs after each search run and were never removed during the reduction process for the WLT and the AE, respectively (see Tables 2, 3 for an overview). Their persistent appearance in the final sets of discriminatory DEGs made them a core set of DEGs that seemed indispensable when distinguishing COPD from control subjects. Notably, the number of persistent DEGs is exponentially reduced with the number of search runs and reaches a number close to 10 or 15 after 7–8 runs (Supplementary Figure S6). We showed that they distinguish COPD from control subjects using PC1 (Fig. 4a, selection subjects) with a resulting AUC of 0.85 and 0.79 for the WLT and the AE, respectively (Fig. 4b). Similar to the 436 and 663 COPD-associated DEGs, no batch effects were observable in PC1 as there is no clear distinction between different comparison groups (Supplementary Figure S2). Moreover, the apparent batch effects we observed in PC2 for the COPD-associated DEGs were further reduced for the persistent genes. Interestingly, only a few of the 25 persistent DEGs have been associated with COPD before (see Discussion and Tables 2, 3).

**Table 2:** 10 persistent DEGs of the WLT. Genes are sorted by the mRNA regulation in COPD and gene symbol (alphabetic).

Name	Gene symbol	mRNA regulation in COPD	Literature evidence supporting a role in COPD
Autophagy related 3	ATG3	Up	Positive regulator of autophagy, involved in TGF-β induced epithelial-to-mesenchymal transition in alveolar epithelial A549 cells^22^
Chimerin 2	CHN2	Up	Activator of RAC1^23^ (see Table 3)
Dihydropyrimidinase	DPYS	Up	Upregulated in response to long-term (9 months) cigarette smoke exposure in mice^24^
Growth arrest specific 2	GAS2	Up	Shown to modulate cell cycle and apoptosis^25^ in a non-COPD context
Gametogenetin	GGN	Up	–
Cysteine rich protein 1	CRIP1	Down	Indirect evidence: CRIP1 acts as carrier for transmucosal zinc absorption^26^. COPD is associated with pulmonary zinc deficiency and in vitro zinc depletion causes bronchial epithelial apoptosis and impairs apoptotic cell clearance^27^
Dipeptidyl peptidase like 6	DPP6	Down	Indirect evidence: DPP6 is a positive regulator of Kv4 potassium channels^28^. Potassium channels are involved in airway smooth muscle contraction^29^. Kv4 channels are also expressed apically by alveolar epithelial cells and are hypothesised to play a role in potassium secretion or oxygen sensing^30^. Structural and functional DPP6 homologue DPP10 is associated with asthma^31^
FRAS1 related extracellular matrix 3	FREM3	Down	Associated with accelerated aging^32^ in a non-COPD context
Polypeptide N-acetylgalactosaminyltransferase 14	GALNT14	Down	–
Pyroglutamylated RFamide peptide receptor	QRFPR	Down	–

**Table 3:** 15 persistent DEGs of the AE.

Name	Gene symbol	mRNA regulation in COPD	Literature evidence supporting a role in COPD
Eva-1 homolog C	EVA1C	Up	–
Glutamate decarboxylase 1	GAD1	Up	Biosynthetic enzyme for neurotransmitter gamma-amino-butyric acid (GABA). Its mRNA expression is upregulated in AE of COPD patients. Also upregulated in healthy smokers and associated with increased epithelial MUC5AC^14,33^
MOB kinase activator 3C	MOB3C	Up	–
Phospholipase C gamma 2	PLCG2	Up	Downstream mediator of RAC1^34^ (see RAC1 below). Gene polymorphisms associated with asthma in southwest European population^35^
Rac family small GTPase 1	RAC1	Up	RAC1 signalling is activated by cigarette smoke and mediates inflammation^36^, oxidative stress, protease (matrix metallaprotease 9) and mucus secretion^37,38^, as well as epithelial-to-mesenchymal-transition^39^. RAC1 is required for airway smooth muscle contraction^40^
Selenoprotein H	SELENOH	Up	–
URB1 ribosome biogenesis homolog	URB1	Up	–
WD repeat domain 4	WDR4	Up	–
Chromosome 10 open reading frame 53	C10orf53	Down	–
Cysteine rich secretory protein 3	CRISP3	Down	–
Integral membrane protein 2A	ITM2A	Down	–
Peroxisomal biogenesis factor 5 like	PEX5L	Down	–
RIPOR family member 3	RIPOR3	Down	–
Secretoglobin family 3A member 2	SCGB3A2	Down	Supports lung development, anti-apoptotic^41^, anti-fibrotic^42^, modulates inflammation (mostly anti-inflammatory)^43,44^. Gene polymorphisms associated with asthma risk in Asian populations^45,46^
Surfactant protein B	SFTPB	Down	Anti-inflammatory^47,48^, promotes alveolar stability. Gene polymorphisms associated with COPD risk, lung function (FEV1), exacerbation frequency and respiratory failure^49–52^. Protein levels decreased in BALF of COPD patients, correlated with lung function^53^

Genes are sorted by the mRNA regulation in COPD and gene symbol (alphabetic).

To validate if the distinction between COPD and control subjects using the 10 and 15 persistent DEGs is reproducible, we tested their predictive capability in three independent gene expression datasets. The first dataset contained WLT samples from a Spanish population comparing 23 CS with COPD to 6 without COPD (GSE103174^19^), while the second one contained SAE samples from 8 FS with COPD and 14 without taken from a Canadian population (GSE56341^20^). The third dataset contained 135 SAE samples taken from an US American population (GSE20257^21^), but as many of them were already included in our comparison group AE3, we only considered 8 CS with COPD and 21 without COPD for validation. All 10 and 15 persistent genes except FRAS1 related extracellular matrix 3 (FREM3, from WLT) were measured in the corresponding validation datasets. To be able to compute the PC1 scores, gene expression values needed to be available for all persistent genes. We therefore imputed values for FREM3 based on the mean expression values of the three WLT gene expression datasets (see Methods). When computing the PC1 scores of the validation subjects, we observed a similar separation between COPD and controls (Fig. 4a, validation subjects) with improved AUC values for both lung sample types (WLT: AUC = 0.91, AE: AUC = 0.85) (Fig. 4b). Of the 25 persistent genes, 20 showed the same trend in regulation as found in our comparison groups of the corresponding lung sample type (Supplementary Figure S7). When adjusted for multiple testing, only one of the observed differences in gene expression was significant (*q* < 0.05) which presumably resulted from the low numbers of subjects.

To investigate possible associations between the persistent DEGs and lung function (i.e. FEV1% predicted) or lung damage (i.e. % emphysema), we applied multiple linear regression analysis to the total study population including both COPD subjects and controls. We analysed the association between persistent gene expression values and FEV1% predicted for both lung sample types, while values for the % emphysema were only available for two WLT comparison groups (WLT2 & 3). As FEV1% predicted and % emphysema exhibited moderate multicollinearity (variance inflation factor (VIF) > 2.0), we ran separate regression models for these factors. Overall, most of the 10 and 15 persistent DEGs showed a significant association (*p* < 0.05) between their expression and FEV1% predicted when adjusted for age, gender, comparison group (only WLT), smoking status (only AE) and smoking history (i.e. pack-years; only AE) (Supplementary Table S3). From the AE, peroxisomal biogenesis factor 5 like (PLCG2) did not display any association with lung function, while WD repeat domain 4 (WDR4) and eva-1 homolog C (EVA1C) showed a significant association (*p* < 0.05) with lung function only in men and MOB kinase activator 3C (MOB3C) only in CS (Supplementary Table S3). The expression of dihydropyrimidinase (DPYS) from the WLT and URB1 ribosome biogenesis homolog (URB1) from the AE showed the strongest association (lowest *p* value) with lung function when adjusted for confounders (Fig. 4c). Their expression both increased with decreasing lung function. The regression analysis on the 10 persistent DEGs from the WLT and % emphysema revealed similar results with 8 genes showing a significant association (*p* < 0.01) when adjusted for age, gender and comparison group. Pyroglutamylated RFamide peptide receptor (QRFPR) did not show any association, while autophagy related 3 (ATG3) was only associated with lung damage in men from WLT2.

## Discussion

In this study, we aimed to identify a small set of robustly COPD-associated genes to extend the knowledge on molecular mechanisms underlying this chronic respiratory disease. To base our analysis on a large sample size, we combined several gene expression datasets containing in total 405 WLT and 411 AE samples from COPD and control subjects. Our focus on transcriptional data from microarray studies restricted the analysis to genes which were measured by the different arrays. The testing for significant DEGs (*p* < 0.05) revealed different numbers for each dataset. To limit their effect on the differences in expression, factors such as gender, age, current smoking status and smoking history were accounted for (see Results). Only DEGs with a consistent expression pattern over all datasets of one lung sample type were selected for further analysis. We showed that these selection criteria resulted in two lists of 436 and 663 COPD-associated DEGs. When examined by PCA, the first principle component accounted for about 30% of the variability in the data and significantly (*p* < 10^–10^) distinguished COPD from control subjects. There remained an overlap between the two subject groups leading to an incomplete separation, likely resulting from uncertainties in the data. Uncertainties affect the expression of genes and arise for instance from differences in the time since smoking cessation or different lifestyles. Such information was unavailable to us and therefore not accountable for in the analysis. Based on our findings, we developed a novel computational approach combining PCA with RDFS to select subsets of COPD-associated DEGs which improve the distinction between COPD and control subjects. In contrast to the study of Mostafaei et al. which uses partly black-box models to select important subsets of genes^14^, our method selects genes based on their performance in distinguishing COPD from control using the first principal component. Our approach reduced the number of 436 and 663 COPD-associated DEGs to about 35 and 66 genes that showed an improved performance in distinguishing COPD from control subjects compared to the initial sets. This confirms that our approach managed to detect smaller subsets of COPD-associated DEGs which improve the distinction. Importantly, the average AUC values between training and test subjects were comparable, showing that the reduced sets performed well also on independent test data. While comparing the different reduced sets of DEGs from each lung sample type, we identified two sets of 10 and 15 DEGs which persistently appeared in all of them. These persistent DEGs not only performed well in distinguishing COPD from control subjects in the original datasets (used to identify the genes), they also showed an improved performance in three independent gene expression datasets including about 20–30 subjects from a Spanish, a Canadian and an US American population, respectively^19–21^. This result suggests that our approach was able to select a core set of DEGs with high predictive capability for COPD in different populations without overfitting to the data used for the identification of the genes. When compared to the study of Mostafaei et al.^14^, only 1 of our 15 persistent DEGs from the AE was among the 44 candidate genes selected for their importance in predicting COPD. This small overlap between the two studies can be explained by differences between the initial sets of genes fed into the selection algorithms (our 663 COPD-associated DEGs vs. 90 genes associated with the progression of COPD), differences in methods used to identify the small sets of genes (our approach combining PCA with a RDFS vs. the combination of several different machine-learning based methods) and differences in sample size (411 in our study vs. 133). Finally, while our study largely rules out overfitting to one specific dataset, this might have contributed to the gene selection of Mostafaei et al. who did not perform external validation^14^. Although only one of the persistent DEGs was significantly differentially expressed (*q* < 0.05) in one of the three independent datasets presumably due to the low numbers of subjects, we showed that similar expression patterns (up- or downregulation) of the persistent DEGs are observable in COPD patients not included for the identification of the DEGs. Additionally, most of the persistent DEGs showed a significant (*p* < 0.05) association with lung function and lung damage when adjusted for confounding factors like age and gender using multiple linear regression models. These findings indicate that our small sets of persistent DEGs could be possible players in the pathogenesis of COPD.

For surfactant protein B (SFTPB, downregulated in AE of COPD patients), a role in COPD pathogenesis was previously established based on genetic polymorphisms ^50–52^, decreased protein concentrations in bronchoalveolar lavage fluid (BALF)^53^ of COPD patients and in vitro experiments^41^. SFTPB promotes alveolar stability by decreasing surface tension of epithelial lining fluid and has been shown to repress macrophage nitric oxide (NO)-mediated inflammation^47^. Thus, it appears to be a protective factor in healthy AE, while being transcriptionally repressed in COPD. Another AE candidate, glutamate decarboxylase 1 (GAD1, upregulated in AE of COPD patients) was among the 44 genes selected by Mostafaei et al. based on its importance in predicting COPD using an AE gene expression dataset^14^. Xiang et al. have shown that expression of GAD1 is increased in AE of persons with asthma and associated with goblet cell hyperplasia and mucus hyperproduction^54^. In another study, in vitro exposure of AE cells to tobacco smoke resulted in increased expression of GAD1 and the mucin MUC5AC, suggesting that GAD1 may also contribute to mucus hyperproduction in smoking-associated COPD^33^. None of the other genes identified here have previously been associated with COPD, although some are known to be involved in biological functions that are relevant for COPD pathogenesis, such as transmucosal zinc absorption (CRIP1^27^), fibrosis (SCGB3A2^41,42^), epithelial-to-mesenchymal transition (RAC1^39^) and inflammation (also RAC1^36^). External validation of changes in protein expression of these genes in lung samples from COPD patients, along with experimental studies, are required to corroborate their role in COPD pathogenesis.

Notably, our analysis is based on gene expression datasets originating only from populations in North America (USA and Canada). We validated the predictive capability and the expression of the 25 persistent DEGs in three independent gene expression datasets from Spain, Canada and the USA, but their number of sampled subjects was limited and a validation in bigger populations from different countries would be necessary to further strengthen their role and the evidence of their consistent change in expression in COPD patients. Additional data would be particularly valuable if linked to subject attributes such as time since smoking cessation. This would enable studying associations between these attributes and the expression of the 25 persistent DEGs.

To conclude, our novel computational approach identified both previously established and novel COPD-associated genes. Identification of previously identified genes supports suitability of our approach for identifying pathophysiologically relevant genes, whereas the novel genes (not previously associated with COPD) extend the available knowledge on molecular mechanisms contributing to lung damage in COPD. For many of these novel genes, a role in COPD pathophysiology is supported by their established cellular functions. Nevertheless, experimental follow-up studies are required to validate the differential expression and potential pathobiological involvement of these genes in the pathogenesis of COPD.

## Methods

### Study design

The aim of this study was to identify a small set of DEGs associated with the observed lung damage in COPD patients. To base our analysis on a large number of subjects, we combined several public gene expression datasets containing two different lung sample types (WLT and AE). We searched the gene expression datasets for COPD-associated DEGs using two selection criteria (see Results) and validated their ability in distinguishing COPD from control subjects using PCA. To afterwards reduce the number of COPD-associated DEGs, we developed a novel approach based on PCA and RDFS. This approach searches for subsets of DEGs that improve the distinction between COPD and control subjects using PCA while randomly removing single genes. The search was run and validated on different compilations of training and test subjects respectively to remove influences of population compositions on the resulting sets of discriminatory DEGs. The sets of DEGs that persistently appeared in all resulting sets of discriminatory DEGs of one lung sample type were considered as the final small set of COPD-associated DEGs. We further validated their expression and their ability in distinguishing COPD from control subjects using PCA in three independent gene expression datasets. We also investigated if their expression associates with lung function or lung damage when adjusted for factors like age, gender and smoking status using multiple linear regression.

### Selection of gene expression datasets

We searched the NCBI Gene Expression Omnibus (GEO) database (https://www.ncbi.nlm.nih.gov/geo/) for human (*Homo sapiens*) datasets matching the search terms “COPD” or “chronic obstructive pulmonary disease”. We included datasets if they (1) contained clinically collected lung samples, (2) compared subjects with no confirmed COPD (controls) to subjects with COPD and (3) contained information about the age, gender, disease status and smoking history of the subjects. We further excluded datasets that had sampled only a small number of subjects (< 9) for at least one of the subject groups. Summary statistics for each included dataset are shown in Table 1.

### Microarray data processing

We downloaded the raw microarray files and the corresponding clinical information file from NCBI GEO. For microarrays from Affymetrix (Human Genome U133 Plus 2.0 Array (GPL570), Human Gene 1.0 ST Array (GPL6244) or Human Genome U219 Array (GPL13667)), we additionally downloaded the CDF files from the Affymetrix Support by Product webpage (https://www.affymetrix.com/support/technical/byproduct.affx) and normalised the intensity values using the Robust Multi-array Average (RMA) procedure. For microarrays from Agilent (Whole Human Genome Microarray 4 × 44 k (GPL6480) or SurePrint G3 Human GE 8 × 60 K Microarray (GPL14550)) or Illumina (HumanHT-12 v4.0 Gene Expression BeadChip (GPL10558)), intensity values were normalised by applying RMA background adjustment, quantile normalisation and log2 transformation consecutively. We performed a quality assessment of all normalised microarrays using boxplots. We did not exclude any microarray. Before further analysis, probes were removed from each dataset if they (1) acted as microarray controls or (2) could not be matched to any Entrez Gene ID. If several probes matched the same Entrez Gene ID, only the one with the highest mean intensity values calculated over all microarrays was considered. Log2 fold changes were calculated by subtracting the mean log2 intensity values of the control from the mean log2 intensity values of the COPD subjects. Significant (*p* < 0.05) differences in the log2 gene expression values between control and COPD subjects were assessed using a two-sample *t*-test. *p* values were adjusted for multiple testing using the Benjamini–Hochberg procedure. We implemented all microarray processing steps using MATLAB (R2019b, The MathWorks, Inc.), its Bioinformatics Toolbox and its Statistics and Machine Learning Toolbox.

### Principal component analysis (PCA)

We used PCA to assess the distinction between COPD and control subjects. For the combined analysis of different datasets, the log2 gene expression values of each comparison group were rescaled to a range between 0 and 1 by the min–max normalisation. The rescaled gene expression values of all subjects were then combined in one dataset for each lung sample type. The PCA was run on the rescaled expression values of the genes mentioned in the corresponding Results parts. For the validation of the persistent DEGs, the log2 gene expression values of the three validation datasets were rescaled as described before. Values for missing genes were imputed by computing the mean of the rescaled log2 gene expression values from the corresponding comparison groups used to select the persistent genes. The means were computed for COPD and control subjects separately. To transform the rescaled persistent gene expression values of the validation dataset using the PCA computed on the corresponding comparison groups, we used the following formula:

$${S}_{val}=\left({X}_{val}-\mu \right)*L$$

with ${S}_{val}$ = PC scores for validation subjects, ${X}_{val}$ = rescaled log2 gene expression values of validation subjects, $\mu$ = estimated mean for gene expressions and $L$ = PC loadings computed on the original comparison groups. To check for a distinction between COPD and control subjects, we only considered PC1. The PCA was applied using MATLAB (R2019b, The MathWorks, Inc.) and its Statistics and Machine Learning Toolbox.

### Random depth-first search (RDFS)

We implemented a RDFS^55^ approach to reduce the number of COPD-associated DEGs while improving the distinction between control and COPD subjects. During the search, random single genes get removed and the performance of the remaining set of DEGs in distinguishing COPD from control subjects is assessed using PCA and the *p* value resulting from a two-sample *t*-test. We used the *t*-test since it considers both the mean and the variance of the PC1 scores when computing the test statistics. A decrease in the *p* value can originate from an increase in distance between the two means or a decrease in variance (or both), two events that improve the distinction between COPD and control subjects. The process of our search approach is shown in Fig. 3a and a pseudo-code of our recursive implementation can be found in the Supplementary Information. We implemented the RDFS using MATLAB (R2019b, The MathWorks, Inc.) and its Statistics and Machine Learning Toolbox.

We ran the RDFS for each lung sample type 10 times with 200 iterations. For each of these 10 runs, a new set of test and training subjects was compiled. The test subjects were randomly selected (10% of all subjects) and only used to assess the performance of the resulting sets of discriminatory DEGs. The remaining 90% of all subjects served as training subjects and were used during the search. For each of these runs, we obtained as many sets of discriminatory DEGs as we ran iterations. The one with the lowest *p* value was then chosen as the final set of discriminatory DEGs for this run. Due to the random nature of our approach (random selection of training and test subjects and random removal of single genes), not every run led to the same set of discriminatory DEGs.

### Receiver operating characteristic (ROC) curves

The ROC curves were computed on the rescaled PC1 scores and compared the true positive rate (TPR) with the false positive rate (FPR) using variable PC1 thresholds. ROC curves and the resulting area under the ROC curve (AUC) give a general estimate of the performance of a set of DEGs in distinguishing COPD from control subjects. For choosing a fixed threshold to discriminate, we used the F-score. The F-score represents the harmonic mean between the TPR and the rate of true positives over all predicted positives known as positive predictive value (PPV). A value of 1 indicates perfect TPR and PPV values using the current threshold. The F-scores were calculated on the training subjects only. All performance measures were computed using MATLAB (R2019b, The MathWorks, Inc.) and its Statistics and Machine Learning Toolbox.

### Multiple linear regression analysis

We tested if the gene expression of the two persistent sets of DEGs can be associated to lung function (FEV1% predicted) or lung damage (% emphysema) using multiple linear regression. The different subject characteristics (e.g. age, gender, FEV1% predicted) served as independent variables and gene expression values as dependent variable for the regression models. Categorical variables were coded as one or several dichotomous variables (e.g. gender: male = 1, female = 0). Before we ran the multiple linear regression analyses, independent variables were investigated for multicollinearity using the VIF and for effect modification using stratified analysis. Collinear variables are not independent of each other, and we only included one of them in our regression models. If categorical variables were effect modifiers (i.e. the different categories led to different effects in association between lung function or lung damage and gene expression), we ran separate regression analyses for the different categories. We only considered a categorical variable as effect modifier if the association between lung function/lung damage and gene expression was significant (*p* < 0.05) for at least one category. We performed the multiple linear regression analyses using MATLAB (R2019b, The MathWorks, Inc.).

### Statistical analysis

Differences in age, pack-years and FEV1% predicted distributions between COPD and control subjects of a comparison group were tested using a two-sample *t*-test, while differences in gender distributions were tested using a Fisher’s exact test. If the age or gender of subjects in a dataset were unknown, we used the statistical values reported in the original publication. A Lilliefors test was used to assess if the PC1 scores of COPD and control subjects followed a normal distribution. If this hypothesis had to be rejected (*p* < 0.05), a Mann–Whitney U-test was used to further assess if there was a significant difference (*p* < 0.05) between the two groups. If not, a two-sample *t*-test was used. We made an exception for the RDFS where we assumed normal distribution and always used a two-sample *t*-test to standardise the comparison. All statistical analysis was performed using MATLAB (R2019b, The MathWorks, Inc.) and its Statistics and Machine Learning Toolbox. If not stated differentially, values are shown as mean ± standard deviation (SD).

## Supplementary Information

Supplementary Information.

Supplementary Tables.

## Data Availability

All gene expression datasets analysed in this study are freely accessible at NCBI GEO (https://www.ncbi.nlm.nih.gov/geo/) with accession numbers GSE11906, GSE20257, GSE37147, GSE47460, GSE56341, GSE64614, GSE76925 and GSE103174.

## Abbreviations

COPD: Chronic obstructive pulmonary disease
WLT: Whole lung tissue
AE: Airway epithelium
SAE: Small airway epithelium
FS: Former smoker
CS: Current smoker
DEG: Differentially expressed gene
PCA: Principal component analysis
PC1: Principal component 1
RDFS: Random depth-first search
SD: Standard deviation
SEM: Standard error of the mean

## Code availability

Our implementation of the RDFS is available on the webpage of the corresponding author (https://www.ntnu.edu/chemeng/research/cybergenome-lab) or can be made available by the corresponding author upon reasonable request.
