# Supplementary material for: Novel computational analysis of large transcriptome datasets identifies sets of genes distinguishing chronic obstructive pulmonary disease from healthy lung samples
Source: Sci Rep. 2021 May 13;11:10258. doi: 10.1038/s41598-021-89762-8 (PMC8119951; doi:10.1038/s41598-021-89762-8)
Supplement: Supplementary file 1 — Supplementary Information. [file 41598_2021_89762_MOESM1_ESM.pdf]

Novel computational analysis of large transcriptome datasets identifies sets of genes distinguishing chronic obstructive pulmonary disease from healthy lung samples

Fabienne K. Roessler[1], Birke J. Benedikter[2,3], Bernd Schmeck[2,4,5,6], Nadav Bar[1]

[1] Department of Chemical Engineering, Norwegian University of Science and Technology (NTNU), Trondheim, Norway

[2] Institute for Lung Research, Universities of Giessen and Marburg Lung Centre, Philipps University Marburg, Marburg, Germany

[3] Department of Medical Microbiology, Maastricht University Medical Center (MUMC+), Maastricht, The Netherlands

[4] Department of Pulmonary and Critical Care Medicine, University Medical Center Marburg, Universities of Giessen and Marburg Lung Center, Philipps University Marburg, Hesse, Germany

[5] Institute for Lung Health (ILH), Giessen, Germany

[6] Member of the German Center for Lung Research (DZL), the German Center for Infection Research (DZIF), and the Center for Synthetic Microbiology (SYNMIKRO) Marburg, Hesse, Germany

Corresponding author: **Nadav Bar**, nadi.bar@ntnu.no

Content of supplementary information

	Page
• Pseudo-code of random depth-first search	3
• Supplementary Figure S1	4
• Supplementary Figure S2	5
• Supplementary Figure S3	6
• Supplementary Table S1	7
• Supplementary Figure S4	8
• Supplementary Table S2	9
• Supplementary Figure S5	10
• Supplementary Figure S6	11
• Supplementary Figure S7	12

Algorithm 1. Recursive implementation of random depth-first search

function *run*():
    G = Gene expression matrix of initial set of COPD-associated DEGs
    PC = Apply PCA to G
    p = Calculate *p*-value using *t*-test on PC(1) of controls vs. PC(1) of COPD subjects
    for i = 1 : iterations **do**
        red_DEGs(i) **=** *random_depth_first_search*(G, p)
    end
end

function *random_depth_first_search*(G_{init}, p_{init}):
    G_{red} = Remove 1 gene randomly from G_{init}
    PC = Apply PCA to G_{red}
    p_{red} = Calculate *p*-value using *t*-test on PC(1) of controls vs. PC(1) of COPD subjects
    if $p_{red} < p_{init}$ **do**
        red_DEGs = *random_depth_first_search*(G_{red}, p_{red})
    else do
        if all genes have been removed and tested **do**
            red_DEGs = read gene IDs from G_{init}
            return
        else do
            red_DEGs = *random_depth_first_search*(G_{init}, p_{init})
    end
end

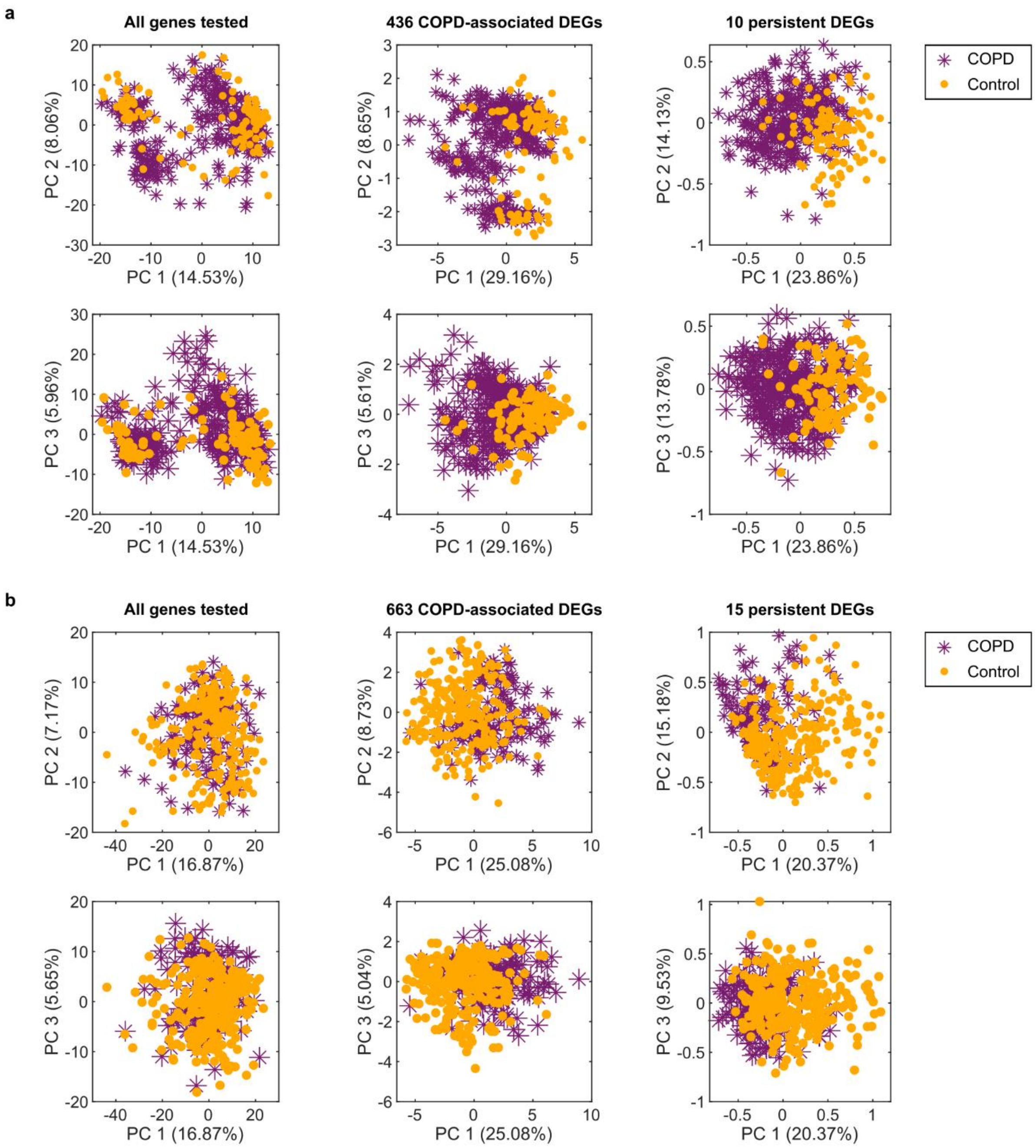

Supplementary Figure S1. PCA plots for different sets of genes from the (**a**) WLT and the (**b**) AE. The PCA was applied to the rescaled expression values of the respective genes named in the title. The percentage of total explained variance by each principal component (PC) is shown in brackets.

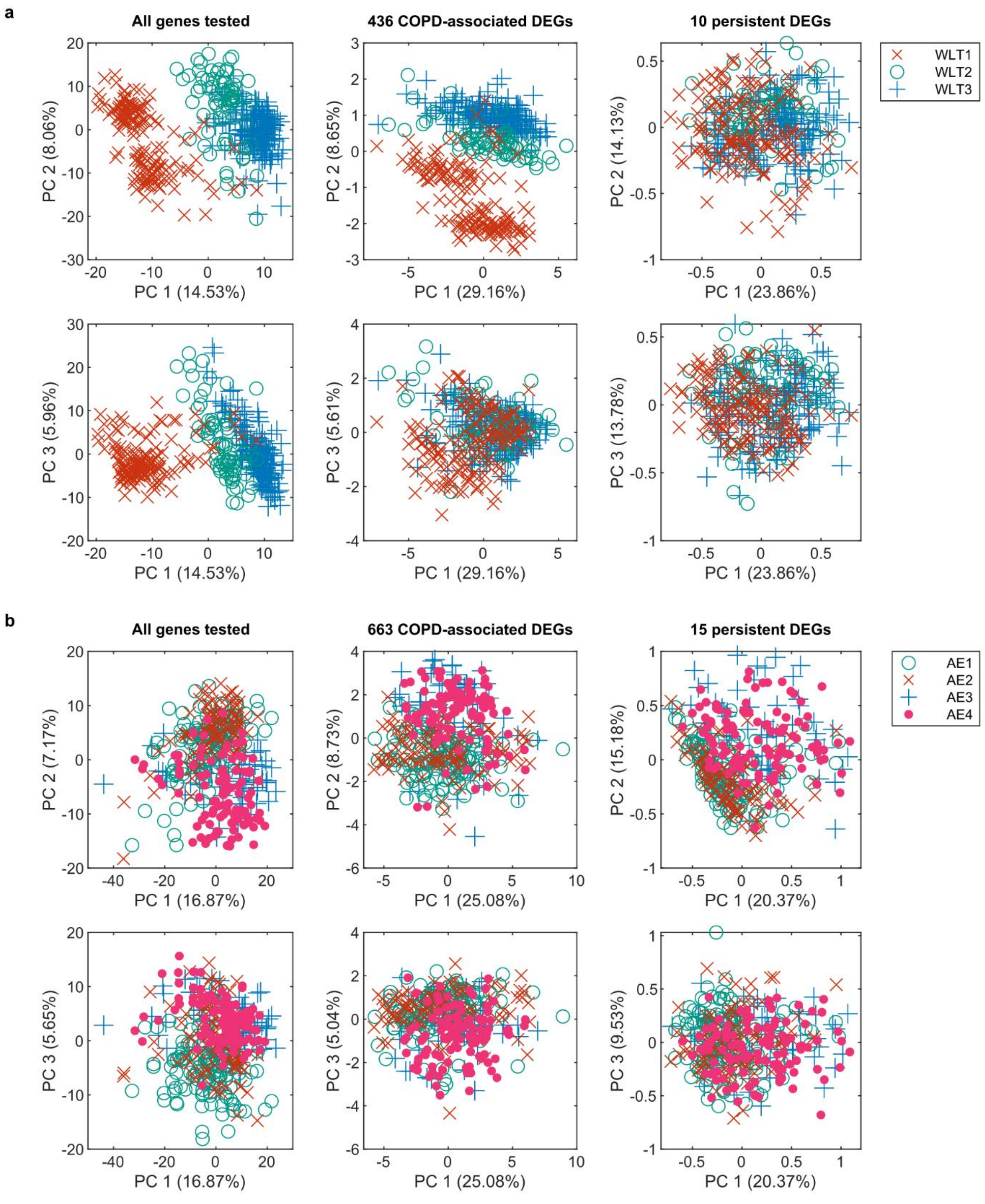

Supplementary Figure S2. PCA plots for different sets of genes from the (**a**) WLT and the (**b**) AE. In contrast to Supplementary Figure S1, single subjects are labelled for their corresponding comparison group (WLT1-3 or AE1-4).

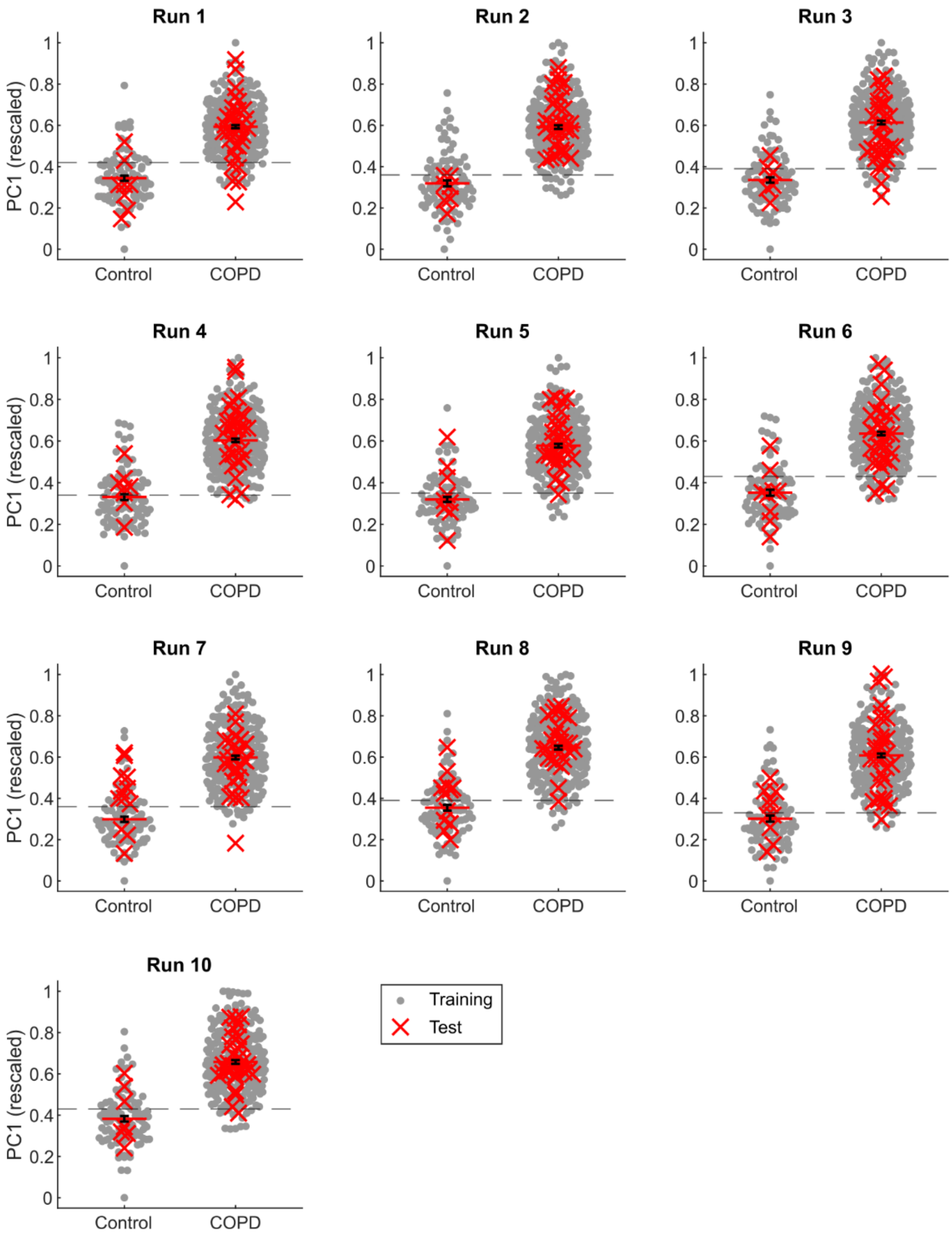

Supplementary Figure S3. Beeswarm plots comparing PC1 scores computed on the rescaled expression values of the different sets of discriminatory DEGs from the WLT. The sets of discriminatory DEGs were selected by our RDFS based on ten different compilations of training subjects (= 10 different runs). Test subjects were only used for validation. The dashed line represents the PC1 threshold with the highest F-score. Red lines with black error bars show the mean ± SEM.

Supplementary Table S1. Results of the RDFS on 436 COPD-associated DEGs from WLT.

Run	Number of discriminatory DEGs	*p*-value	PC1 threshold	TPR		TNR		AUC	
				Train	Test	Train	Test	Train	Test
1	37	$2.05*10^{-47}$	0.42	0.93	0.84	0.80	0.75	0.92	0.93
2	37	$3.69*10^{-45}$	0.36	0.96	1.00	0.68	1.00	0.91	1.00
3	37	$1.48*10^{-49}$	0.39	0.96	0.91	0.74	0.71	0.93	0.93
4	37	$1.90*10^{-47}$	0.34	0.99	0.94	0.60	0.25	0.92	0.90
5	32	$1.23*10^{-45}$	0.35	0.96	0.97	0.69	0.67	0.93	0.91
6	26	$4.23*10^{-48}$	0.43	0.94	0.90	0.79	0.78	0.92	0.93
7	38	$4.27*10^{-51}$	0.36	0.96	0.96	0.76	0.25	0.94	0.80
8	35	$3.98*10^{-46}$	0.39	0.97	0.96	0.67	0.42	0.92	0.93
9	28	$3.97*10^{-50}$	0.33	0.98	0.97	0.66	0.50	0.93	0.89
10	46	$8.62*10^{-46}$	0.43	0.96	0.97	0.71	0.57	0.92	0.94
**Mean (± SD)**	**35 (± 5.62)**	**$6.22*10^{-46}$ (± $1.16*10^{-45}$)**	**0.38 (± 0.04)**	**0.96 (± 0.02)**	**0.94 (± 0.05)**	**0.71 (± 0.06)**	**0.59 (± 0.24)**	**0.92 (± 0.01)**	**0.92 (± 0.05)**

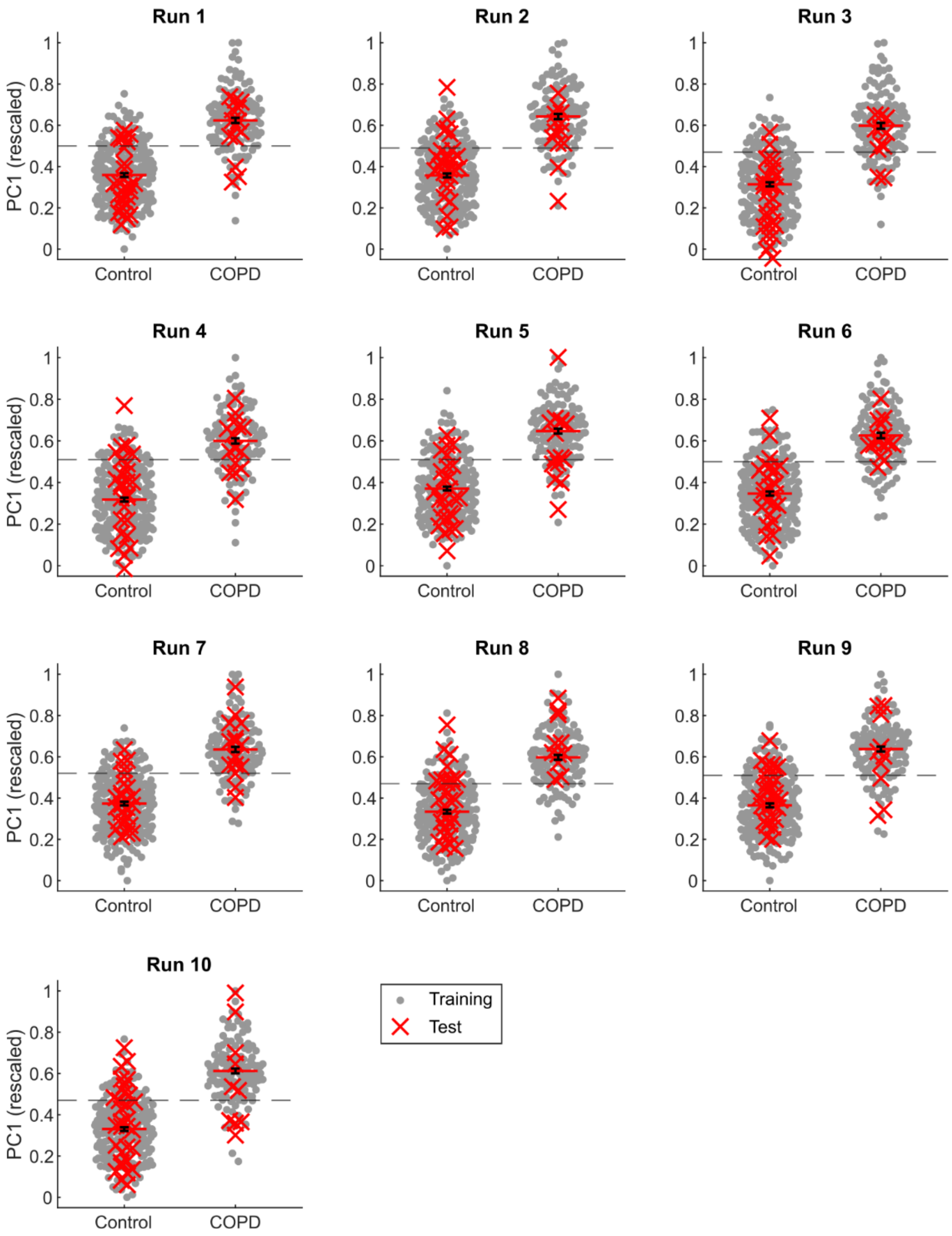

Supplementary Figure S4. Beeswarm plots comparing PC1 scores computed on the rescaled expression values of the different sets of discriminatory DEGs from the AE. The sets of discriminatory DEGs were selected by our RDFS based on ten different compilations of training subjects (= 10 different runs). Test subjects were only used for validation. The dashed line represents the PC1 threshold with the highest F-score. Red lines with black error bars show the mean ± SEM.

Supplementary Table S2. Results of the RDFS on 663 COPD-associated DEGs from AE.

Run	Number of discriminatory DEGs	*p*-value	PC1 threshold	TPR		TNR		AUC	
				Train	Test	Train	Test	Train	Test
1	70	$4.87*10^{-49}$	0.50	0.84	0.79	0.84	0.78	0.91	0.91
2	67	$3.49*10^{-53}$	0.49	0.88	0.85	0.82	0.82	0.92	0.81
3	65	$2.67*10^{-48}$	0.47	0.81	0.83	0.85	0.93	0.90	0.93
4	63	$3.34*10^{-50}$	0.51	0.80	0.67	0.88	0.81	0.91	0.84
5	57	$4.58*10^{-53}$	0.51	0.88	0.67	0.83	0.81	0.92	0.84
6	67	$3.43*10^{-49}$	0.50	0.84	0.93	0.84	0.89	0.91	0.92
7	63	$1.66*10^{-48}$	0.52	0.84	0.81	0.85	0.84	0.91	0.92
8	69	$3.02*10^{-50}$	0.47	0.86	1.00	0.85	0.63	0.91	0.93
9	63	$6.35*10^{-52}$	0.51	0.85	0.70	0.84	0.74	0.91	0.80
10	71	$1.42*10^{-54}$	0.47	0.90	0.60	0.84	0.68	0.92	0.75
**Mean (± SD)**	**66 (± 4.20)**	**$5.22*10^{-49}$ (± $9.14*10^{-49}$)**	**0.50 (± 0.02)**	**0.85 (± 0.03)**	**0.78 (± 0.13)**	**0.84 (± 0.02)**	**0.79 (± 0.09)**	**0.91 (± 0.01)**	**0.87 (± 0.07)**

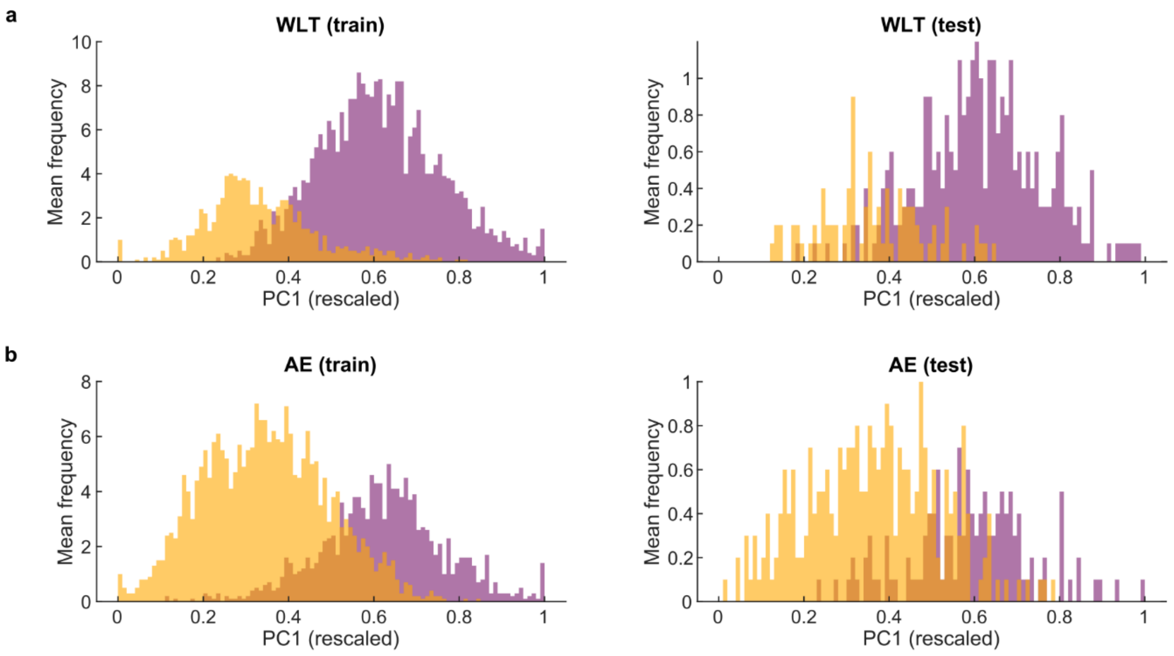

Supplementary Figure S5. Histograms showing the mean frequency of rescaled PC1 scores for COPD and control subjects over all ten search runs (see Figure 3b for smoothed histograms). Rescaled PC1 scores for each set of discriminatory DEGs are shown in Supplementary Figure S2 and S3. (**a**) shows the distribution of PC1 scores for training and test subjects sampled from the WLT, while (**b**) shows them for the AE.

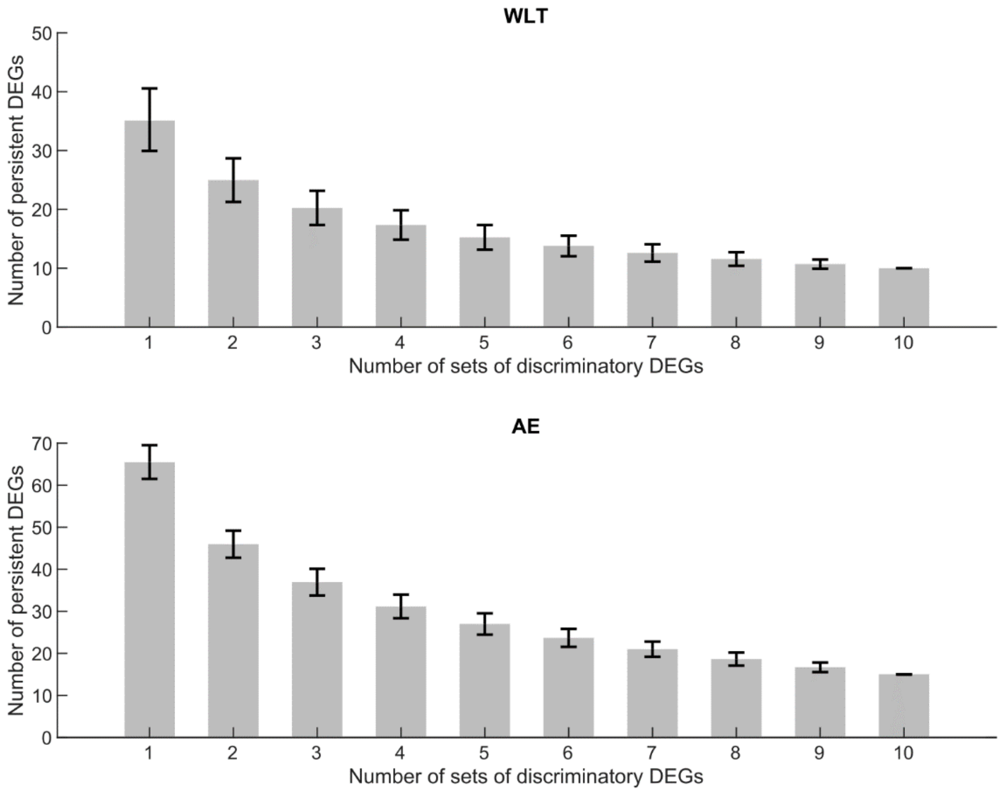

Supplementary Figure S6. Bar diagram showing the reduction in number of overlapping DEGs (= persistent DEGs) with increasing numbers of sets of discriminatory DEGs. The bars were computed on different orders of sets of discriminatory DEGs to account for the influence of their order in appearance on the numbers of persistent DEGs. We see that the number of persistent DEGs almost stagnates after 7-8 runs. Error bars show the SD.

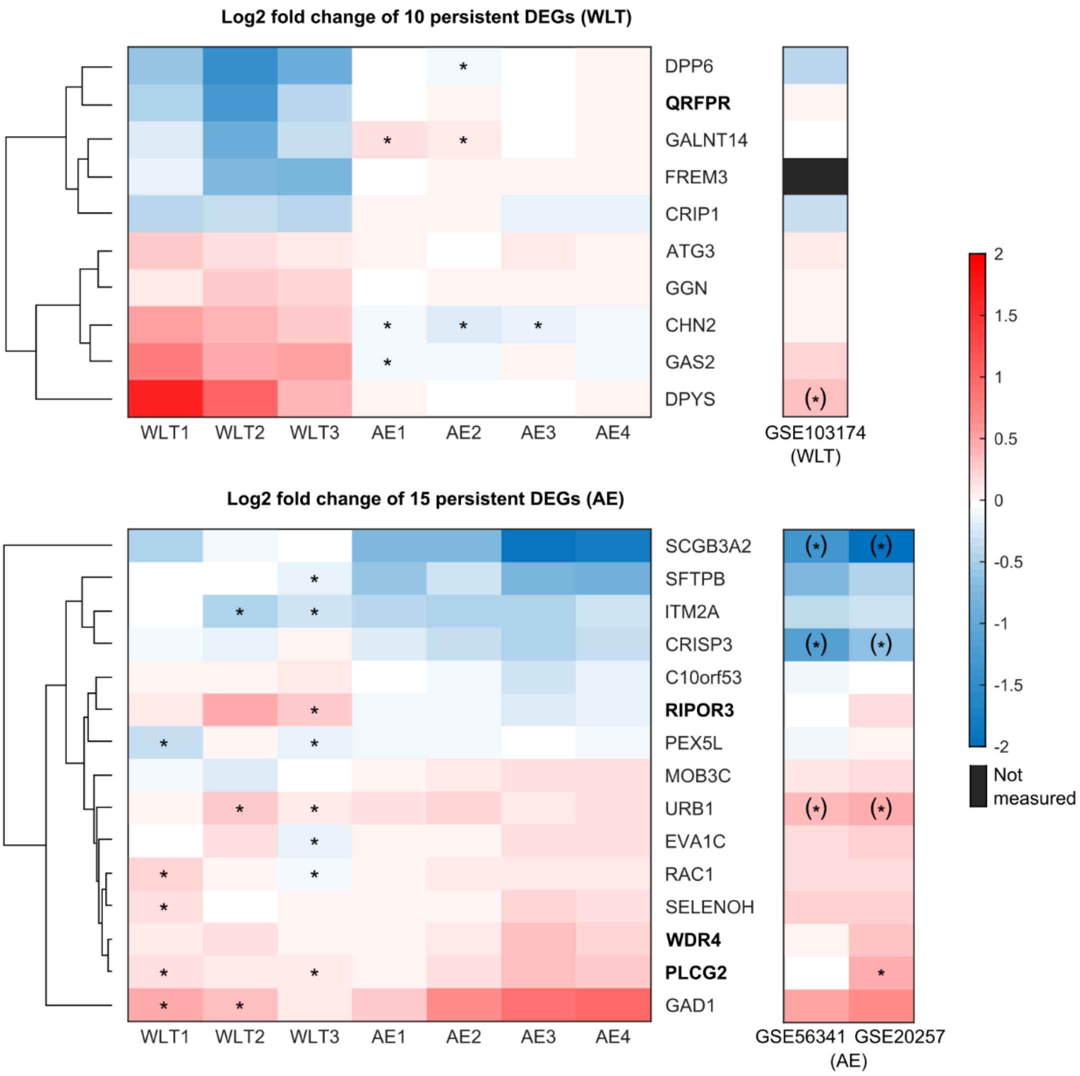

Supplementary Figure S7. Heatmaps showing the hierarchical clustering of the 10 and 15 persistent DEGs based on their log2 fold change measured in the seven comparison groups (WLT1-3 and AE1-4). The log2 fold changes measured in three independent datasets (GSE103174, GSE56341 and GSE20257) are displayed to the right. **Bold** printed gene symbols indicate that these genes did not show the same sign in fold change in one of the independent datasets compared to the comparison groups the DEG originates from (e.g. PLCG2 from AE1-4). Asterisks * show if expression values in COPD subjects were significantly different (p<0.05) from control subjects (only shown for the lung sample type the DEG does not originate from and the independent datasets for reasons of clarity). For the independent datasets, brackets around the asterisks (*) show if the expression values were not significantly different (q<0.05) anymore after adjustment for multiple testing.
